# Supplementary material for: Factors contributing to immunization coverage among children less than 5 years in Nadowli-Kaleo District of Upper West Region, Ghana
Source: PLOS Glob Public Health. 2024 Aug 1;4(8):e0002881. doi: 10.1371/journal.pgph.0002881 (PMC11293730; doi:10.1371/journal.pgph.0002881)
Supplement: Attachment — Submitted filename: Response to Reviewers.docx [file pgph.0002881.s003.docx]

Robert Kogi

P.O. Box 22

Kukuom

08/06/2024

The Editor

PLOS Global Public Health

Dear Editor,

**Response to Reviewers Comments**

I am happy to bring to your notice that, I have responded to all the comments made on our manuscript. The responses to the individual comments are appropriately reported in the table below for your consideration.

Thank you.

Sincerely,

Robert Kogi.

| **Comments** | **Responses** |
| --- | --- |
| **Journal Requirements** | |
| 1. In the ethics statement in the Methods, you have specified that verbal consent was obtained. Please provide additional details regarding how this consent was documented and witnessed, and state whether this was approved by the IRB. | I have addressed this as captured in lines 218-225 |
| **Reviewer #1** | |
| Title and abstract  Lines 20-21: – Here, the authors should qualify the immunization status e.g., fully immunized status  Line 29 – Nothing was said about immunization coverage in the abstract hence the conclusion should be on fully immunization status e.g., coverage for children with fully immunized status is not very encouraging | #1. “fully immunized status” has been added in line 23.  #2. This is addressed and captured in lines 32-33 as “coverage for children with fully immunized status is not very encouraging at our study site” |
| Background  Lines 80-82: – Probable over the past five years would make it clearer. | In lines 121-122, the comment was addressed as “…the immunization coverage has been consistently below the national target of 95% coverage for all antigens over the past five years” |
| Methods  Sample Size Determination  Lines 155-156: - Some minor correction needed here in the word used to begin the sentence, | This was addressed as in line 155 as “We calculated the sample size for this study using the Cochran formula…” |
| Sampling method  Lines 179-180: - To remove the word ‘be’, | The word “be” has been deleted in lines 165-166 |
| Ethical Issues  Lines 213-214: - Upper West Regional Directorate Should be Upper West Regional Health Directorate. | In lines 215-217, this comment was addressed as “Permission was sought from the Upper West Regional Health Directorate and the District Health Directorate of the Nadowli-Kaleo District.” |
| Lines 222-223: - The word ‘were’ should be removed for the sentence to read ‘Research assistants and participants also used alcohol-based hand sanitizers throughout the period of the data collection. | This has been addressed in lines 230 and 231 |
| Results Lines 231-232: - First born should be firstborns or written with a hyphen.  Lines 237-238: - Kindly add monthly if this is their monthly earnings.  Table 2 title is not complete – Should be Socio-demographic characteristics of mothers/caregivers of children under 5 years in Upper west Region, Ghana  Lines 246-248: - The concluding part should be rephrased to respondents whose children were fully immunized were within 31-35 years so that it does not make it looks like you are referring to immunization status of the mothers or caretakers.  Table 3 Association between respondents’ socio-demographic characteristics and child full immunization – Child full immunization should be changed to immunization status since the table is looking at both full and partial immunization status.  Lines 274-276:– late during immunization should be late during immunization sessions or services.  Lines 276-277: - Add centres or sessions to immunization – e.g. mothers who spent less than 30 minutes in immunization centres or sessions.  Table 6 – According to literature the term predictors require a more statistically robust analysis and hence the authors could change predictors to factors associated with immunization status. | #1. In line 240, the phrase “first born” is changed to “first-born”  #2. In lines 245-246, this comment is addressed as “In addition, less than two-fifth (44.1%) of the respondents earned from Gh₵100.00- <Gh₵500.00 monthly.”  #3. In lines 249-250, this comment is addressed as “Socio-demographic characteristics of mothers/caregivers of children under 5 years in Nadowli-Kaleo District, Upper West Region, Ghana”  #4. This comment is effected in lines 255-256  #5. In lines 273-274, this comment is addressed as “Association between respondents’ socio-demographic characteristics and child immunization status”  #6. This comment is addressed and captured in lines 285 and 285  The correction to this comment can be found in lines 286 and 286  #7. The title of Table 6 is revised in lines 300 and 301 as “Factors associated with immunization status among children under five years old in Nadowli-Kaleo District” |
| Discussion Lines 301-302: - The word ‘our’ study should be removed and immunized fully should be fully immunized. Also, I would replace majority with a little over half of respondents.  Line 302: - I would say this present study’s coverage of fully immunized children is lower, compared to the Ghana national…  Lines 305-306 – 77.4 % cannot be lower than 55.5% so kindly correct the sentence  Line 315 – Add years to 19 i-e 19years and above  Lines 345-346 – The authors are comparing a child born in a health facility to those delivered at home but the last part of the sentence doesn’t make it look like the comparison is with home deliveries.  Lines 349-352 – Kindly check the sentence again Lines 355-357 – should be during immunization sessions or services Lines 362-364 – Should be immunization centres  Line 376 – I would suggest to rephrase as in conclusion, the immunization status of fully immunized children…. Line 385 – Included should be including | #1. Revision to this comment was done in line 309  #2. Revision to this comment was done in line 310  #3. This sentence was corrected and captured in lines 312-314  #4. This is corrected in line 325  #5. This comment has been addressed in lines 346-347 as “This supported the finding from Kenya where a child born in a health facility was five times more likely to be vaccinated than a child delivered at home [5]”  #6. This is corrected in lines 351-353  #7. This is corrected in lines 356-357  #8. This is addressed in line 360  #9. This is addressed in line 375  #10. This is addressed in line 384 |
| Acknowledgement: - Authors could use passive voice or use we/us in place of I/my. | This is addressed in lines 393-396 |
| **Reviewer #2** | |
| 1. There is no scientific writing where the main objective or objectives to be achieved would not be stated. Even though authors' background to the study and literature were clear enough. One can easily state their objectives on their behalf but that is not done. The objectives to be achieved in this article was CONSCUPIOUSLY MISSING! This is not acceptable in any scientific writing no matter how it has been implied in the background to the study. This needs to be clearly stated somewhere towards the end of the background to the study. | This comment is addressed in lines 126-127. |
| 2. Similarly, this article ended with the discussion section. Every scientific article must have a conclusion section where findings are summarised and a conclusion is drawn. This again was missing from this article. Drawing conclusions and making recommendations within discussion section does not go well with scientific writing of this nature. | #1. A summary of the study is added in the manuscript from lines 363-375  #2. A conclusion on the study was added and captured in lines 376-385 |
| 3. Many results were repeated inside the discussion even with statistical data again which also is not good. In standard discussion, you discuss the important or significant results vis a vis existing studies and not necessarily repeating your results all over again. Then, you engage existing studies in comparison with your findings not just saying that your study agree or disagree with a particular study. This is not enough. | The entire discussion section of the study is revised taking into cognisant this comment. This is captured from lines 309-362 |
| 4. Your recommendations were mere pieces of advice which did not emanate from findings from your study. Authors should know the differences between suggestions and recommendations. Your recommendations should emanate from your findings and not just stating things you feel should be done. | This comment is addressed in lines 385-390 |
| 5. It is surprising that the authors were reporting data in the range of 40-50% of the respondents and called that "the majority" and at the same time reporting those in the region of 70% and above still as the majority. Please look into this. Data that are less than 50% of the total population of the respondents should never be reported as the majority. For instance, data in the region of 50-55% of the total population could be called " more than half", "above average" and so on while you report those that are more than 80% of the total population as the majority. Authors should seriously look into this. | These comments made in the results section of the manuscript have been addressed in the entire Results section from lines 235 to 298 |
| 6. In modern statistical reporting and as a rule of thumb, it is not okay to report both absolute numbers and percentages in the same study. You either concentrate on reporting absolute numbers or percentages. In most cases, reporting percentages is better in scientific articles of this nature. This is also APA 6th and 7th editions standard. | The reporting of the study’s results has been revised to include only percentages as recommended by the reviewer, and captured in lines 235-298 |
| 7. In the last statement of the abstract, authors stated that they "suggest". In scientific writing, you don't suggest. You kindly recommend based on the outcome of your results, not just mere suggestions for the sake of it | This comment is addressed in lines 33-35 as “To enhance childhood full immunization, we recommend expansion of access to maternal and child health services, encouraging institutional childbirth, and timely regular antenatal visits.” |
| 8. Writing a good background to the study requires some technicality. it is better if the authors highlight all the evidences or factors affecting immunization uptake or coverage from developing countries before finally ending the background to the study with the situation in Ghana since that is their main focus. It is not good enough if you have spoken about Ghana before ending your background to the study with reports from another country before stating your objectives | The background of the study has been restructured to align with this comment, and captured in lines 69-125 |
| - This is an incomplete statement. Disease outbreak among who? Children or adults? Increased costs on what? Treating infant diseases or?  - Get the data for immunization coverage for the entirre Nadowli-Kaleo district before determining whether it is low or otherwise  - Check this regression result again. P-value can't be 2.12. I guess you were trying to write the entire value of AOR, CI and p-value together. Do it well if this is the case  - Authors need to be careful of incomplete sentences and phrases. For instance, who is this unfavourable attitudes referring to? Caregivers or immunization officers or who? It is not clear enough.  -Although your sample size determination was not based on this population, but it is still important to give a clear update of what the population of the district should be in 2024. So, I suggest you use arithmetic or geometric method to do the population projection of the district from 2010 when the last population and housing census was done till 2024 using the prevailing annual growth rate of rate  - Where was it imported from? Excel sheet? SPSS? Make this known.  - There is repetition here about obtaining consent from participants | #1. This is addressed and captured in lines 9-11  #2. This is addressed in lines 12-14  #3. This is addressed line 29 and 298  #4. This comment is addressed line 94  #5. This comment is addressed lines 140-142  #6.This comment is addressed and captured line 200  #7. This comment is addressed lines 217-222 |
